# Thinking differently about integration: people-centred care and the role of local communities

**DOI:** 10.5334/ijic.1736

**Published:** 2014-09-25

**Authors:** Nick Goodwin

**Affiliations:** International Journal of Integrated Care

Integrated care represents an approach to the delivery of services that seeks to coordinate care both with and around the needs of service users, their families and the communities to which they belong. There is considerable evidence that the use of such a perspective needs to lie at the heart of any strategy or discussion about integrated care [1]. It helps to provide a unifying narrative for the purpose of integration and a compelling logic as to its core objectives and how innovations should be judged [2]. It also helps shift the focus away from the tendency to focus on technological, structural and organisational approaches to integration that may not necessarily achieve better care that is coordinated around people's needs.

Being person-centred requires care services to be responsive to a person's holistic needs (and goals) that emerge from their own personal social determinants of health. In other words, care services should become tailored to meet an individual's or group's specific characteristics and potential within the context of their lives. Though commitment to such approaches varies, strategies such as coproducing care plans and care packages with service users, educational programmes that support health literacy and self-care and the enablement of independence through assisted living are common strategies within well-functioning integrated care programmes.

However, extending the principle of coproduction to the community-level has received less attention. The idea is that people-centred care should go beyond an approach that confronts common epidemiological population profiles (e.g., through population health management using risk stratification techniques) to one that considers the holistic needs and aims of the community in an evolutionary movement that should strengthen communities’ competencies and action towards health and well-being.

The people-centred approach is more public health-focused and places a greater emphasis on quality of life as a more meaningful outcome than quality of care. This is important since studies in care to people with both physical and mental health comorbidities demonstrate that more effective approaches are those that focus on quality of life and enabling people to live well with their conditions [3, 4]. To achieve this, strategies focusing on emotional and physical well-being, interpersonal relationships, personal development, and self-determination are required. Such approaches typically involve non-professional care where members of the local community are a key resource in care delivery.

The importance of communities as coproducers of integrated care has been recently articulated in this journal by Henk Nies [5] based upon a presentation at our 14^th^ International Conference on Integrated Care held in Brussels last April. Nies challenges us to ‘think differently’ about integrated care since, in his view, the term has become too much a professionals’ concept in research, theory and practice and that the paradigms of integrated care, especially to those with complex needs, should be reconsidered. Specifically, Nies makes three key arguments:

1. That the current concept of health as the ‘absence of disease’ is outdated when it should be better conceived as the ‘ability to adapt and self-manage in the face of social, physical and emotional challenges’ so requiring a focus on strengthening capabilities, resilience and physical and mental well-being.
2. That quality of life, rather than quality of care, is now a more meaningful outcome to people living with long-term health problems (as discussed above).
3. That population-based approaches to integrated care in which local communities are involved in the codesign and delivery of care promote initiatives that support self-care as well as create community responsibility for health.

The arguments of Henk Nies are more than a working hypothesis. There is good evidence to demonstrate the value of community engagement to support healthier living [6], whilst other evidence on care to people with complex needs has highlighted the centrality of local communities, including the voluntary sector, as part of a successful integrated care ecosystem [7]. Building community awareness and trust with local populations as a strategy is a long-term process that transfers power (and risk) to the community and requires a culturally sensitive approach.

A good example of what can be achieved through such an approach can be found in the Nuka Health System in Alaska where health care to the indigenous community has been significantly improved through developing community-owned and integrated health care solutions [8]. Led by a clear mission for *working together with the Native Community to achieve wellness through health and related services* the Alaskan Native people took over the ownership and management of their health system and established a range of primary care centres offering a wide range of inter-disciplinary services to support people's health and wellbeing. Active ownership of the Nuka Health System by the local community has been central to the achievement of successful outcomes including increased enrolment in primary care; elimination of waiting lists for behavioural health consultations; increased patient satisfaction and significant reductions in the use of specialist and hospital-based treatment.

As Nies suggests, our understanding of integrated care needs to incorporate the recognition that communities are coproducers of health and well-being. The current segregation of professional and non-professional care in practice, therefore, needs to be addressed since it represents a unresolved issue of care fragmentation. Nies’ challenge, therefore, is a fundamental one since it asks us to re-evaluate the extent to which integrated care can truly fulfil the principles of person- and people-centred care that form part of its definition and central philosophy.

Coproducing care with patients, citizens and communities is a key theme at our Second World Congress on Integrated Care in Sydney on 23–25 November 2014 and 15th International Conference on Integrated Care in Edinburgh on 25–27 March 2015. For more information and to register visit the IFIC website at: http://www.integratedcarefoundation.org/conferences-and-events

## References

[1] Shaw S, Rosen R, Rumbold B (2011). What is integrated care?.

[2] Ham C, Walsh N (2013). Making integrated care happen at scale and pace: lessons from experience.

[3] Smith S, Soubhi H, Fortin M, Hudon C, O'Dowd T (2012). Managing patients with multimorbidity: systematic review of interventions in primary care and community care settings. British Medical Journal.

[4] Schalock RL, Verdugo-Alonso ML (2002). Handbook on quality of life for human services practitioners.

[5] Nies H (2014). Communities as co-producers in integrated care. International Journal of Integrated Care.

[6] Bloomfield M, Cayton H (2009). Community engagement report for the Health Foundation.

[7] Goodwin N, Sonola L, Thiel V, Kodner D (2013). Co-ordinated care for people with complex chronic conditions. Key lessons and markers for success.

[8] Gottlieb K (2013). The Nuka System of Care: improving health through ownership and relationships. International Journal of Circumpolar Health.

